# Perforation of the Meckel’s diverticulum with a chicken bone: a case report and literature review

**DOI:** 10.1186/s40792-019-0577-x

**Published:** 2019-02-01

**Authors:** Bardia Bidarmaghz, Hugh McGregor, Kasra Raufian, Chin Li Tee

**Affiliations:** Department of Surgery, Redcliffe Hospital, Anzac Avenue, Redcliffe, QLD 4020 Australia

**Keywords:** Meckel’s diverticulum, Rule of twos, Foreign object

## Abstract

**Background:**

Meckel’s diverticulum (MD) is the most common congenital abnormality of the gastrointestinal (GI) tract. Most of the people remain asymptomatic during their lifetime, but some can develop complications such as inflammation, haemorrhage or obstruction. Perforation of Meckel’s diverticulum is very rare, and we present a case of perforation by a chicken bone.

**Case presentation:**

A 19-year-old man presented to the emergency department with abdominal pain, and based on examination and laboratory findings, he was diagnosed with appendicitis initially. Meanwhile, a CT scan was requested and a foreign object inside the Meckel’s diverticulum was noted, and on further questioning, he mentioned that he had chicken with bones 2 days ago. He was taken to the operating theatre, and during laparotomy, the perforated Meckel’s diverticulum was found, and it was resected with primary anastomosis.

**Conclusion:**

Meckel’s diverticulum follows the ‘rule of twos’, and perforation of it with foreign object is rare. Patients usually present with signs and symptoms of acute abdomen, and appendicitis is the first diagnosis, and the final diagnosis is usually made intraoperatively. Perforation of Meckel’s diverticulum should be considered for the patients who present with acute abdomen, and with precise history taking and investigations, preoperative diagnosis can be achieved.

## Introduction

Meckel’s diverticulum was first described by Fabricius Hildanus in 1958 and was named after the German anatomist, John Friedrich Meckel, who established its embryological origin in 1809 [1]. It is the most common congenital abnormality of the gastrointestinal (GI) tract with the incidence of 2.2% [2]. It results from the failure of obliteration of the omphalomesenteric duct on the antimesenteric border of the terminal ileum that normally disappears between weeks 6 and 8 of pregnancy [3]. Most people with Meckel’s diverticulum remain asymptomatic during their life, and only 4% develop complications [4]. The most common complications are inflammation, haemorrhage, and obstruction [5]. Perforation of the Meckel’s diverticulum by a foreign object is very rare, and we present a case of a young patient who presented with a Meckel’s diverticulum perforated by a chicken bone.

## Care presentation

**Fig. 1:**
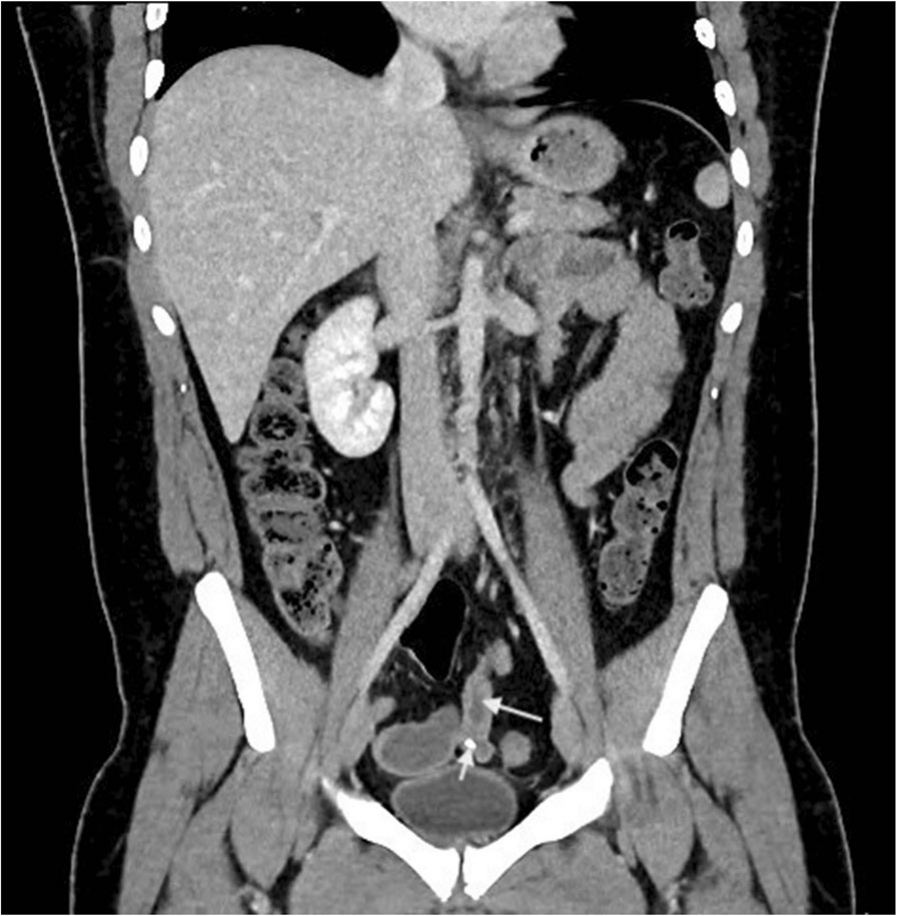
CT coronal view shows chicken bone (arrow head) inside MD (arrow)

**Fig. 2:**
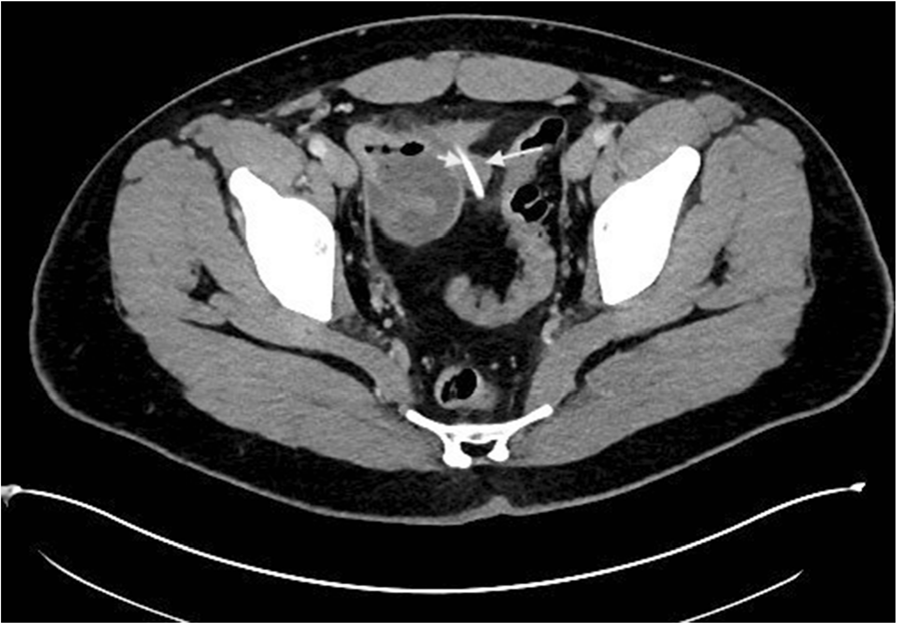
CT transverse view shows chicken bone (arrow head) inside MD (arrow)

**Fig. 3:**
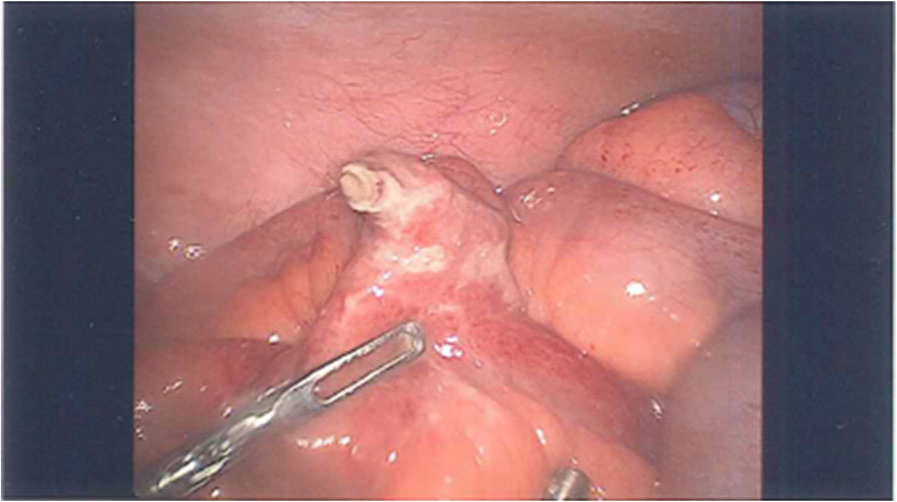
Laparoscopic view of the perforated MD by chicken bone

**Fig. 4:**
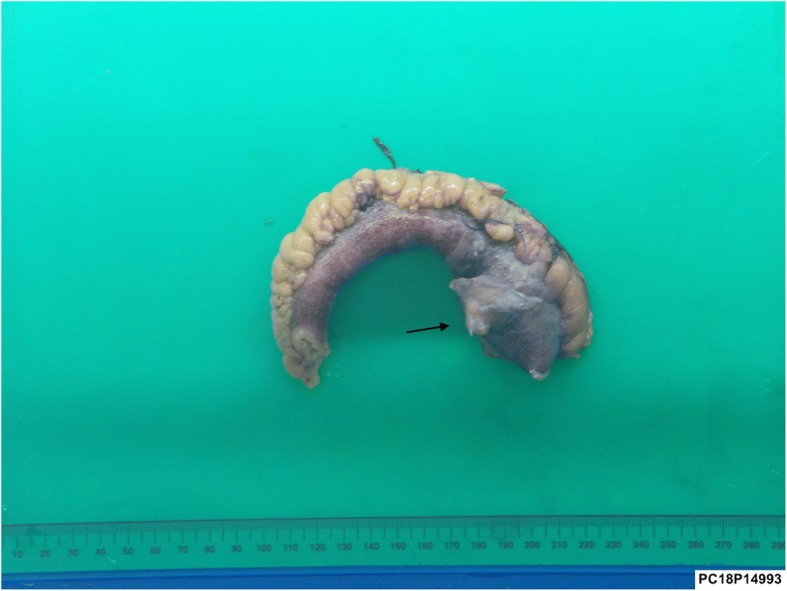
Macroscopic view of the resected bowel containing MD with sign of perforation by chicken bone (arrow)

**Fig. 5:**
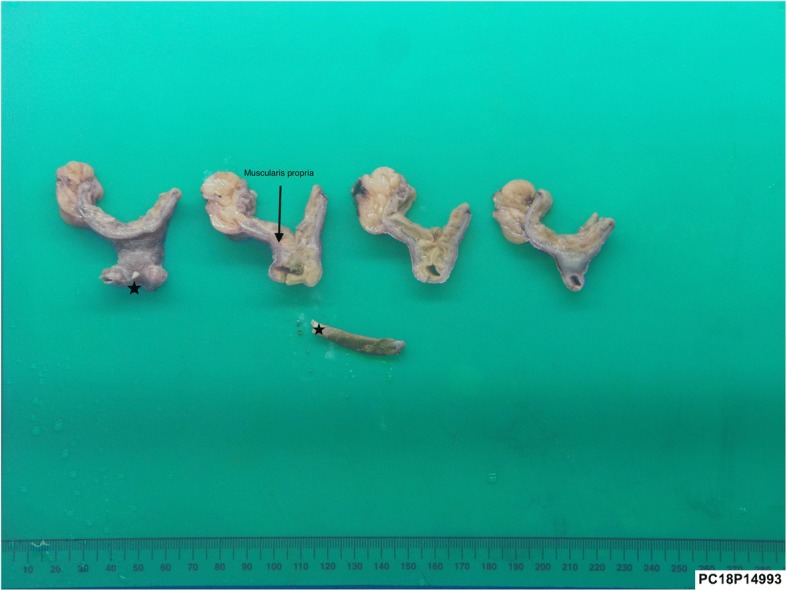
Macro-photos of the diverticulum in cut sections, perforated by the chicken bone (asterisk)

A previously fit and healthy 19-year-old man presented to the emergency department with a 1-day history of crampy lower abdominal pain. He described the pain as intermittent without radiation which was aggravated by eating and relieved by lying flat. He also had subjective fever but no nausea or vomiting and denied genitourinary symptoms. On arrival, the patient was haemodynamically stable with a temperature of 36.3, pulse rate of 87, respiratory rate of 14, blood pressure of 127/77 and oxygen saturation of 96% on room air. On examination, his abdomen was soft without guarding and he was generally tender in the lower part of the abdomen with no sign of peritonism. Laboratory data showed a white cell count of 15.6 × 10^9^/L with neutrophils of 12.29 × 10^9^/L and a C-reactive protein of 50 mg/L. The rest of the electrolytes were normal. The first impression was appendicitis, but because of patient’s atypical presentation and findings, the decision was made to perform a contrast CT scan. This revealed a 35-mm radio opaque foreign body in a small bowel diverticulum with surrounding inflammatory change (Figs. 1 and 2). On further questioning, the patient mentioned that 2 days prior he ate chicken with bones. He was taken to the operating theatre, and during laparoscopy, a chicken bone was found trapped inside the Meckel’s diverticulum. There were signs of impending perforation (Fig. 3). The operation was converted to a lower midline laparotomy. After proper manual assessment of the small bowel, inflammatory wall thickening of 10 cm of the small bowel proximal and distal to the Meckel’s diverticulum area was noted. Decision was made to perform bowel resection, 20 cm of the small bowel loop was resected, and a side to side hand sewn anastomosis was performed (Figs. 4 and 5).

**Fig. 6:**
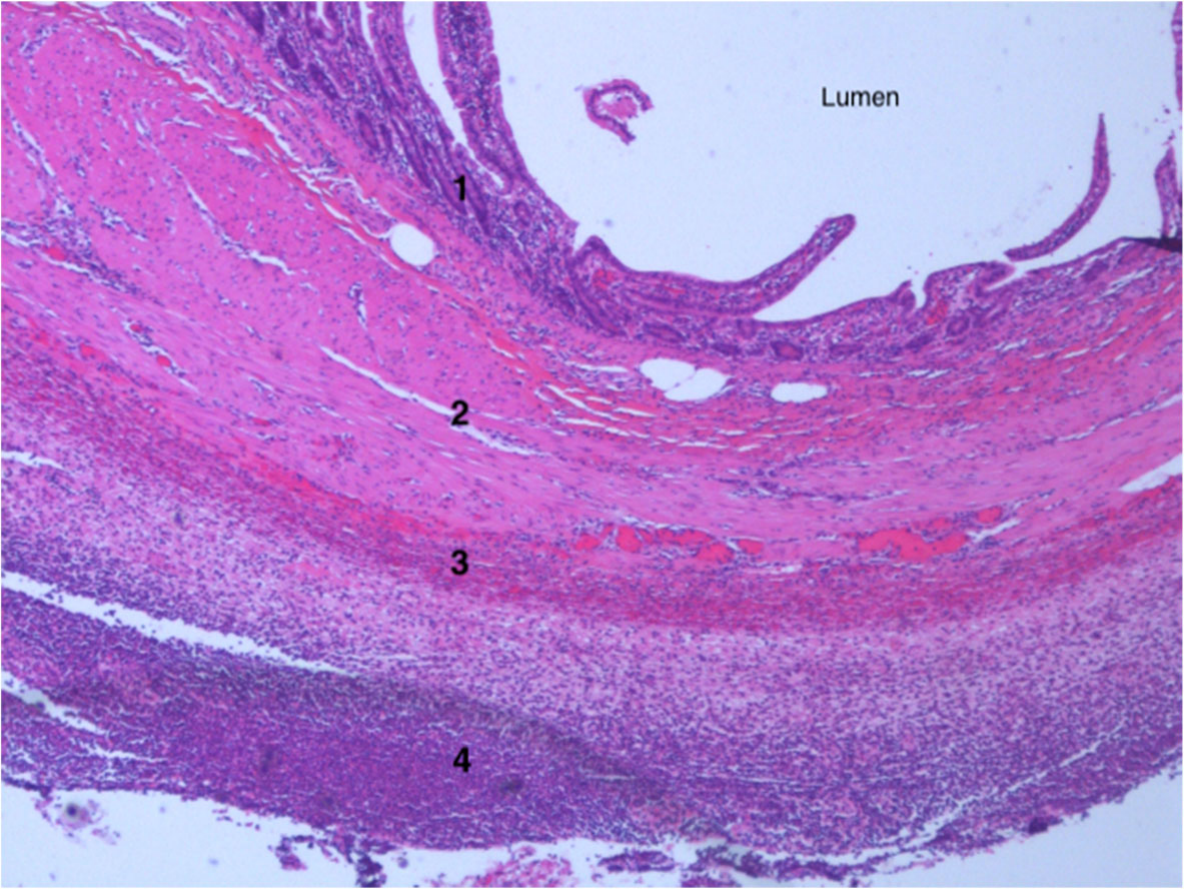
Low power photomicrograph of the diverticulum immediately adjacent to the site of perforation with layers of mucosa (1), submucosa (2), muscularis propria (3) and acute serositis of the adventitia (4)

After the operation, the patient was transferred to the surgical ward and had an uneventful recovery. He was discharged on day 5 of the admission and was later seen in outpatient clinic and had fully recovered. The histopathology demonstrated a small bowel diverticulum with intramural foreign body (chicken bone) and overlying acute faecal peritonitis consistent with microscopic perforation (Fig. 6).

## Discussion

Meckel’s diverticulum is the most common malformation of the GI tract and is present equally in both sexes. It is a true diverticulum as it has all three layers of the small intestine. Meckel’s diverticulum historically follows the ‘rule of twos’: It is found in 2% of the population, usually 2 ft proximal to the ileocaecal valve, 2 in. in length, and more common before the age of 2 and has 2 types of heterotopic mucosa [6]. It is reported that in 55% of Meckel’s diverticula, heterotopic mucosa of gastric and pancreatic tissue is present with the incidence of 60–85% and 5–16%, respectively [7].

The most common complication of a Meckel’s diverticulum among the paediatric population is bleeding secondary to the ulceration by acid secretion from ectopic gastric mucosa. Inflammation, obstruction and intussusception are more common in adult population [8].

It is reported that less than 1% of ingested foreign bodies perforate the GI tract and they are usually sharp or more than 6.5 cm [9]. Perforation of the Meckel’s diverticulum is rare and reported only in 1.6% of the total GI tract perforations secondary to foreign bodies [10]. In 2009, Chan et al. reported 300 cases of perforation of Meckel’s diverticulum by foreign object [6], and among them, cases of wood splinter [11], button battery [12] and chicken and fish bone [6] have been mentioned in literature. It is said that tendency of these objects to lodge in the blind pouch of the Meckel’s diverticulum causes the perforation [13].

Preoperative diagnosis can be challenging as most of the patients do not recall the ingestion of the foreign object [1]. Patients usually present with signs and symptoms of acute abdomen and appendicitis, or perforation of small bowel is the initial diagnosis, and patient is taken to the operating theatre, and accurate diagnosis is made intraoperatively [14]; however in a few cases [15] like ours, careful evaluation of the CT scan leads to preoperative diagnosis of perforation of the Meckel’s diverticulum with foreign object.

Surgery is the mainstay of the treatment. Definite surgical management of the perforated Meckel’s diverticulum varies case to case. If the inflammation and perforation is localised to the diverticulum, simple diverticulectomy with wedge resection or tangential resection and repair in a horizontal fashion for avoidance of stricture formation can be achieved [16]. Bowel resection is indicated when an inflammatory or ischaemic process involves the adjacent ileum [17]. In our case, as the inflammation and wall thickening had already spread to the proximal and distal part of the ileum, decision was made for resection of the affected segment with primary handsewn anastomosis.

## Conclusion

Although it is a rare condition, perforation of the Meckel’s diverticulum by foreign object must be suspected in patients who present with acute abdomen. Most of the cases are diagnosed intraoperatively, but careful history and review of the scan findings can yield to preoperative diagnosis.

## Abbreviations

CT: Computed tomography
GI: Gastrointestinal
MD: Meckel’s diverticulum
